# Investigation of the esthetic perception of different canine parameters

**DOI:** 10.1007/s00784-022-04651-2

**Published:** 2022-07-29

**Authors:** Niklas Schwefer, Sandra Freitag-Wolf, Gunnar Meyer, Matthias Kern

**Affiliations:** 1 Department of Prosthodontics, Propaedeutics and Dental Materials, University Medical Center Schleswig-Holstein, Campus Kiel, Arnold-Heller-Str. 16, 24105 Kiel, Germany; 2 Institute of Medical Informatics and Statistics, University Medical Center Schleswig-Holstein, Campus Kiel, Brunswiker Str. 10, 24105 Kiel, Germany

**Keywords:** Esthetics, Canine morphology, Tooth shade, Crown length, Canine inclination, Angle of incisal edge tip

## Abstract

**Objectives:**

The aim of this study is to analyze the esthetic perception of selected canine features, namely crown length, shade, inclination, and angle of incisal edge tip.

**Materials and methods:**

The anterior maxillary teeth of a Central European woman were photographed and digitally modified in order to investigate esthetic perceptions of the above four categories. Three groups of examiners with different levels of experience in the field of dentistry (laypersons/inexperienced dental students, advanced dental students, dentists) evaluated the photographs twice with the help of visual analogue scales.

**Results:**

The best-evaluated canines have approximately the same length as the central incisor, have the same shade as the other anterior teeth, are best embedded in a lighter overall tooth shade, are neutral to slightly palatal inclined, and have a right angled to rounded incisal edge (≥ 90°). The canines evaluated as least esthetic, however, are longer than the central incisors, darker, inclined labially, and have a tapered incisal edge. No significant differences could be found between the evaluations of the groups with regard to the four feature categories.

**Conclusions:**

Laypersons, advanced dental students, and dentists generally evaluate according to the same esthetic standards. Gender does not have a significant influence on evaluation. Clear definitions of esthetically favored shades, incisal edge shapes, inclination, and lengths of the canines can be given.

**Clinical relevance:**

Since the esthetics of the smile line play a critical role for patients, dentists, dental technicians, and their supplying industry, knowledge of the esthetically preferred morphology of canines is essential.

**Clinical significance:**

The aim of this study is to give clear definitions of esthetically favored shades, incisal edge shapes, and lengths of the canines, as the esthetics of the smile line play a critical role for patients, dentists, dental technicians, and their supplying industry (e.g., denture tooth manufacturers). Precise knowledge of esthetic preferences is important in clinical practice for both dentists and dental technicians, for example, in order to adequately advise patients regarding esthetic corrections. Also, in the case of missing teeth, this knowledge is essential for optimal and satisfactory restorations. Thus, this study can contribute to the satisfaction of general practitioners and patients.

## Introduction

Human canines are an integral part of smiling and are important for the overall esthetics of a smile [1, 2]. Esthetical analyses that consider attractiveness and focus on human canine morphology are rarely found in the literature. Most of the time, such studies focus on maxillary incisal teeth, soft tissues, or only the central incisors [3–7]. Other studies only discuss the role of the esthetic characteristics of the canines as substitutes for the lateral incisors [8–12]. Furthermore, hardly any mathematical tooth proportions (such as the golden proportion, golden percentage) are applicable to the canines [13]. This study examines the esthetically favored shade, incisal edge shape, and crown length of the canines because these features lack clear definitions. Esthetic preferences regarding the inclination of canines have already been evaluated [2, 14]; they are to be verified and validated in this study.

It is also useful to compare different levels of knowledge and experience. As in similar studies that evaluated esthetic perception, the evaluations of laypersons/inexperienced dental students, advanced dental students, and professional dentists will be compared [15, 16].

The objective of this study is to analyze the esthetic perception of selected maxillary canine features in photographs. The shade, shape, inclination, and length of the canines were changed by computer-based manipulation. Our hypothesis is that changing the selected parameters of the canines will result in altered esthetic perception of the smile.

## Materials and methods

A positive vote of the local university ethics committee was obtained (AZ D 508/16).

**Fig.1:**
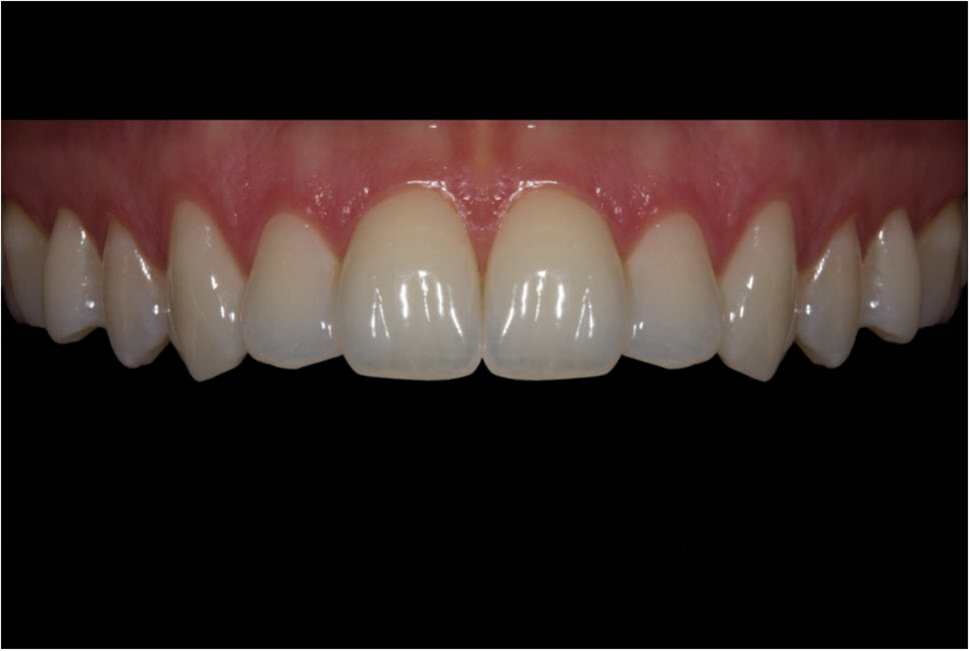
Example of a photograph used in this study (“uniform tooth shade”)

The anterior teeth of a Central European woman were digitally photographed. This woman was selected because her maxillary anterior teeth and the surrounding tissues were healthy and free of restorations and other pathologies that could negatively affect esthetics. Minor visual defects (e.g., enamel cracks, saliva bubbles) in the color photograph were carefully optimized using a professional image-editing program (Photoshop v. 13.0, Adobe Systems Incorporated, San José, USA), preserving the true shape and shade of the teeth (see Fig. 1). The surrounding soft tissues were retracted with cheek retractors to clearly reveal the maxillary teeth up to and including the second premolars. The mandibular teeth played only a minor role in this study and were therefore hidden behind a black cover. In order to unify the right and left sides, the features of only one quadrant of the maxilla were modified and this was then mirrored on the opposite side.

Based on the original photograph, a set of images was created and divided into four categories, each of which focused on one specific feature: (1) crown length of the canines, (2) tooth shade, (3) inclination of the canines, and (4) angle of incisal edge tip.

Twenty different images were divided into these four categories and were compared by three groups of examiners who volunteered without pay: laypersons (*n* = 47), advanced dental students (*n* = 40), and experienced dentists (*n* = 30). In the course of a preliminary study with four volunteer participants and 34 test images, parameter estimands for a sample size calculation could be assumed. To detect differences between the effects of the five settings (manipulated parameters of the size of a onefold standard deviation by an ANOVA with a power of 80%), a group size of 25 is needed, when all four main parameter (angle of incisal edge tip, tooth shade, inclination, length of canines) are considered separately. To ensure that this sample size is reached, we added 5 examiners per group.

The group of laypersons/inexperienced dental students consisted of first-semester dentistry students who had no clinical experience and had no instructions on dental anatomy up to this point. The group of advanced students consisted of dentistry students in the 8th semester or higher. The dentists were employed at the university hospitals in Kiel and Aachen. Within these groups, the examiners were also split according to gender. The survey took place in the classrooms of the universities of Kiel and Aachen.

Before the participants evaluated the images, a standardized mental state questionnaire was used to determine their mental state and thus to ensure the validity of their decisions [17].

The survey was conducted using questionnaires we developed. These were given to the groups at the start of the survey and included visual analogue scales (VAS) for rating attractiveness of the dentition. The left end-point of a line measuring 100 mm was defined as “0 = unesthetic” and the right end-point was defined as “100 = esthetic.” The participants were aware of the basic theme of the work with the main focus on canine morphology, but not of the particular way of manipulating the images.

In order to reduce intra-observer variability, each image was shown twice so that the overall number of images was 40 (2 × 20). The feature categories were randomly mixed, and transitions between slides during the presentation via a beamer were overlaid in black to prevent any direct comparison. In addition, two short, 30-s breaks were taken during the evaluation, which took about 10 min in total. The images were shown via projector in the seminar rooms/lecture halls of the Universities of Kiel and Aachen under adapted lighting conditions.

A total of 117 participants (44 men, 73 women) rated 2 × 20 photographs, which resulted in a total of 4680 evaluations. After averaging the pairs, 2340 evaluations remained for analysis.

### Crown length of canines

**Fig. 2:**
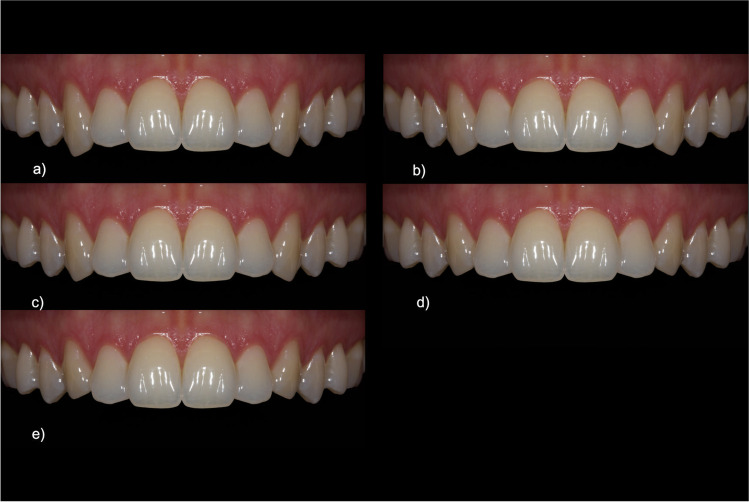
Image set for “crown length of canines”: **a** 1:1.02 cent. inc., **b** 1:1 cent. inc., **c** 1:0.95 cent. inc., **d** 1:1.05 lat. inc., **e** 1:1 lat. inc

The first category was “crown length of canines”. For this, the maxillary canine of one quadrant was first measured digitally on the original image and then modified in relation to the maxillary incisor on the same side. By changing the length of the canine multiple times, five new lengths in relation to the central and lateral incisors were created (1:1.02 cent. inc.; 1:1 cent. inc.; 1:0.95 cent. inc.; 1:1.05 lat. inc.; 1:1 lat. inc.). The quadrant was then mirrored as described above (cf. Figure 2). The ratios were chosen to be within a range that takes into account the natural limitations of dentition.

### Tooth shade

The clinically determined tooth shade corresponded to A1 (VITA classical, VITA Zahnfabrik, Bad Säckingen, Germany). Since exposure time, lighting conditions, color fidelity of monitors, and other technical factors influence the color presentation of the final images, there may be variations in tooth shade between the images rendered on projectors or monitors and the original images. For this reason, VITAPAN classical tooth shade coding was indirectly compared to the tooth shade of the subject via digital luminance measurement in Photoshop, the professional image-editing program we used. Luminance is a measure of the brightness distribution in an image using a dimensionless numerical value. Using Photoshop’s histogram function, corresponding luminance values were determined for the tooth shades and put in relation to the actual tooth shade (A1 in this case). We edited the images according to the luminance changes of the Vitapan classical tooth shades scale. On average, luminance changed by 3.5% from one tooth shade to the next.

**Fig. 3:**
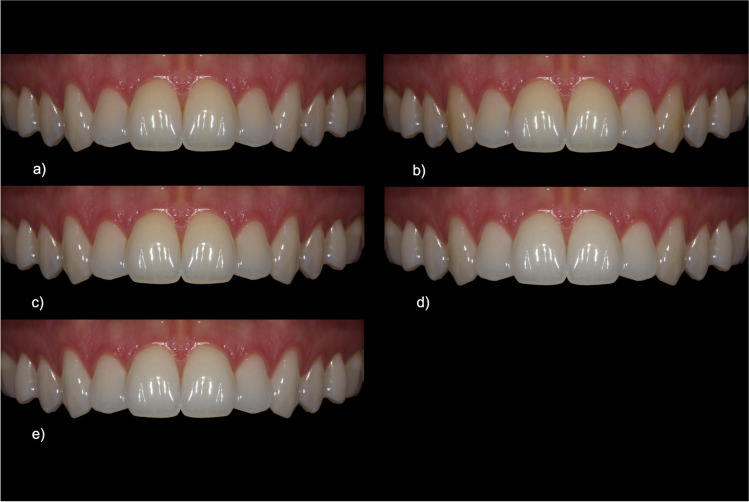
Image set for the category “tooth shade”: **a** darkened anterior teeth + adapted canine, **b** darkened anterior teeth, **c** uniform tooth shade, **d** brightened anterior teeth, **e** brightened anterior teeth + adapted canine

Initially, we only changed the shade of the canines. They were brightened compared to the original shade. The purpose of this brightening was to optically imitate the shade of the incisal teeth and thus generate a uniform tooth shade for the entire anterior maxillary region (cf. Figure 3c). In a second step of tooth shade manipulation, the entire anterior maxillary region was brightened by one shade compared to the natural original image and then again by one shade compared to the manipulated image of uniform tooth shade. This resulted in two variants of the lighter tooth shade: one with more natural, darker canines (cf. Figure 3d), and one with a uniform tooth shade (cf. Figure 3e). As the tooth shade of the subject already corresponded to A1, the luminance was increased by the calculated mean value of 3.5% between each new tooth shade. A similar procedure was used to achieve the opposite effect and the teeth were darkened by one shade (corresponding to the A2 color coding, cf. Figure 3a and b). In this case, too, there were two image versions with different canine shades.

### Inclination of canines

**Fig. 4:**
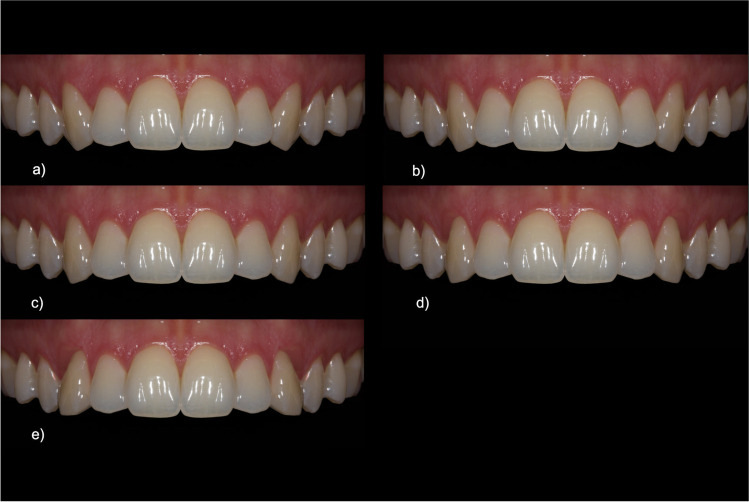
Image set for the category “inclination of canines” (in degrees): **a** − 10, **b** − 5, **c** 0, **d** + 5, **e** + 10

First, the naturally inclined canines were optically straightened, i.e., brought into a non-inclined position in relation to the image axis. Starting from this inclination of 0°, the angle of inclination in labial-palatal direction was changed and then mirrored on the opposite side (cf. Figure 4). Following a comparative study, the naturally occurring angulations of canines in the range from + 10° to − 10° were chosen as reference values [2].

### Angle of the incisal edge tip

**Fig. 5:**
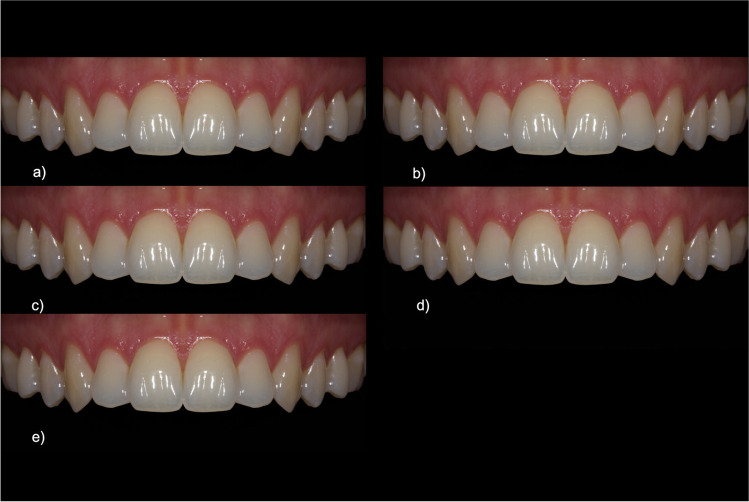
Image set for the category “angle of the incisal edge tip” (in degrees): **a** 100, **b** 95, **c** 90, **d** 85, **e** 80

The category “angle of the incisal edge tip” focused on changes in the shape of the incisal edge of the canines (cf. Figure 5). The angle of the incisal edge (mesial to distal incisal edge) was optically measured in the original image and then changed to previously defined angles (while maintaining the same crown length) from acute to obtuse in order to simulate increasing levels of abrasion. The original angle, which was optically determined using the ruler function in Photoshop, was 94.3°, which was close to the naturally occurring right angle of the average natural canine.

### Statistical analysis

All collected data showed symmetrical distributions, so a normal distribution was assumed and parametric test procedures were used. Duplicate scores were aggregated using means, and these differences were evaluated by *t*-test and correlations analysis. All results are expressed as means with standard deviations and 95% confidence intervals and visualized as Box-and-Whisker plots.

The *t*-test was used for comparisons between independent observations and the paired *t*-test was used for dependent observations. The main effects (angle of incisal edge tip, tooth shade, inclination, length of canines) and two-way interactions were investigated using analysis of variance (ANOVA) following by multiple comparisons using the Scheffé method. The twofold evaluations were randomly analyzed and the Pearson correlation coefficient was calculated.

The SSPS statistics program (IBM v.27, Armonk, New York, USA) was used for statistical analysis and the level of statistical significance was set at 5%.

## Results

Due to a stanine score of 8 or 9 according to the standardized mental state questionnaire, a total of 3 study participants were not evaluated and screened out because of their lowered mood (laypersons 1, advanced dentistry students 2).

As part of the single-stage evaluation, the study participants viewed and evaluated each image twice. The analysis of the two evaluations showed that there were significant differences: the second evaluations were higher than the first (mean 1 50.34 ± 22.8; mean 2 51.19 ± 22.8, *p* = 0.048), which is statistically but not clinically relevant. Moreover, the differences vary widely and the Pearson correlation coefficient was comparable small (*ρ* = 0.581). Therefore, to reduce intra-observer variability and increase accuracy, both assessments were averaged.

The averaged assessment results are presented separately in Table 1 and in Table 2 for the different groups in the category “crown length.” Using the Scheffé method for multiple comparisons, subgroups were formed to show significant differences within the categories. ANOVA (Table 3) showed that there were main effects for all factors (all *p* < 0.001) and one interaction effect between the feature category “crown length of canines” and the groups (*p* = 0.03). All other factors showed no interaction and there were also no significant differences between genders.

**Table 1:** Results. For each manipulated parameter of the categories, the mean ratings, standard deviations, and 95% confidence intervals for *n* = 117 evaluations by different canine parameters are shown

**Parameter**		**Mean** ± **standard deviation** (**CI lower bound/upper bound**)
**Crown length**	1:1 lat. inc.	35.4 ± 18.8 (32.0–38.8)
	1:1.05 lat. inc.	52.0 ± 20.3 (48.3–55.7)
	1:0.95 cent. inc.	57.2 ± 15.7 (54.4–60)
	1:1 cent. inc.	51.2 ± 16.7 (48.2–54.2)
	1:1.02 cent. inc.	23.1 16.3 (20.1–26.1)
**Tooth shade**	darkened anterior teeth + adapted canine	59.1 ± 15.4 (56.3–61.9)
	darkened anterior teeth	53.4 ± 16.2 (50.5–56.3)
	uniform tooth shade	63.5 ± 14.2 (60.9–66.1)
	brightened anterior teeth	59.3 ± 15.3 (56.5–62.1)
	brightened anterior teeth + adapted canine	67.6 ± 16.6 (64.6–70.6)
**Inclination**	− 10	52.7 ± 17.1 (49.6–55.8)
	− 5	56.1 ± 16.0 (53.2–59.0)
	0	58.1 ± 14.9 (55.3–60.8)
	+ 5	51.6 ± 16.8 (48.6–54.6)
	+ 10	23.7 ± 16.9 (20.6–26.8)
**Angle of the incisal edge tip**	80	37.0 ± 18.7 (33.6–40.4)
	85	46.0 ± 17.6 (42.8–49.2)
	90	56.5 ± 16.7 (53.5–59.5)
	95	58.2 ± 15.4 (55.4–61.0)
	100	53.5 ± 15.8 (50.6–56.4)

**Table 2:** Results divided by groups. This table shows the mean ratings, the standard deviations, and the confidence interval (95% level) for each group in the category “crown length of canines”. The number of evaluations for group 1 is *n* = 47, for group 2 *n* = 40, and for group 3 *n* = 30

**Parameter**		**Mean** (**CI lower bound/upper bound**)
**Group 1: laypersons**	1:1 lat. inc.	28.9 ± 15.4 (24.5–33.3)
	1:1.05 lat. inc.	44.2 ± 17.7 (39.1–49.3)
	1:0.95 cent. inc.	51.2 ± 14.5 (47.1–55.3)
	1:1 cent. inc.	50.8 ± 16.9 (46.0–55.6)
	1:1.02 cent. inc.	22.1 ± 15.4 (17.7–26.5)
**Group 2: advanced dentistry students**	1:1 lat. inc.	40.8 ± 20.4 (34.5–47.1)
	1:1.05 lat. inc.	59.4 ± 21.7 (52.7–66.1)
	1:0.95 cent. inc.	61.0 ± 14.6 (56.5–65.5)
	1:1 cent. inc.	54.9 ± 14.3 (50.5–59.3)
	1:1.02 cent. inc.	26.0 ± 17.5 (20.6–31.4)
**Group 3: dentists**	1:1 lat. inc.	38.5 ± 19.0 (31.7–45.3)
	1:1.05 lat. inc.	54.6 ± 18.4 (48.0–61.2)
	1:0.95 cent. inc.	61.6 ± 16.3 (55.8–67.4)
	1:1 cent. inc.	47.1 ± 18.7 (40.4–53.8)
	1:1.02 cent. inc.	21.0 ± 16.2 (15.2–26.8)

**Table 3:** ANOVA. This table shows the results of the ANOVA with main and interaction effects

Dependent variable:	*p*
Angle	< 0.001
Shade	< 0.001
Crown length	< 0.001
Inclination	< 0.001
Group	< 0.001
Gender	0.022
Angle * gender	0.401
Shade * group	0.097
Shade * gender	0.896
Crown length * group	0.030
Crown length * gender	0.176
Inclination * group	0.181
Inclination * gender	0.494

### Crown length of canines

**Fig. 6:**
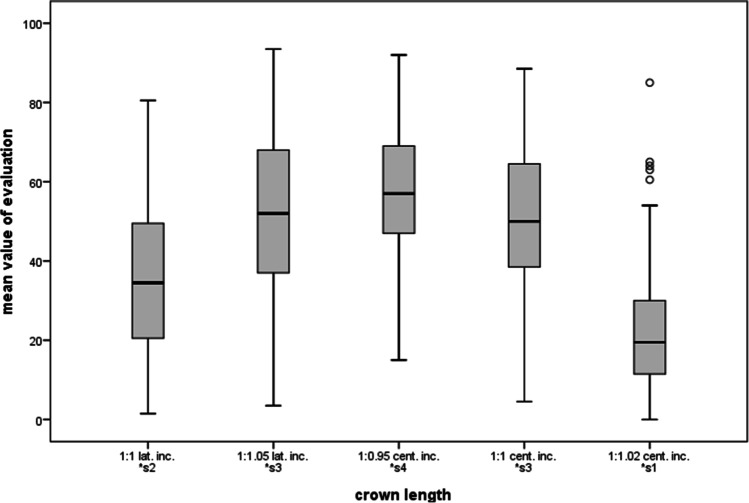
Ratings for the category “crown length of canines” using the visual analogue scale (*y*-axis: 0 corresponds to least, 100 to most esthetic). The *x*-axis indicates the gradations of the feature category, in this case the relative length (in each case with reference to the central [“cent. inc.”] or lateral [“lat. inc.”] incisor) in ascending order. Multiple comparison resulted in the subgroups from s1 to s4 with *p* ≤ 0.05 across subgroups, indicated by *

If the canines were approximately the same length as the central incisors (1:1 cent. inc., 51.2 CI = [48.2–54.2]) or slightly shorter (1:0.95 cent. inc., 7.2 CI = [54.4/60.0], 1:1.05 lat. inc., 52.0 CI = [48.3–55.7]), this was considered most esthetic (see Fig. 6, Table 1). However, if the canines were longer (1:1.02 cent. inc., 23.1 CI = [20.1–26.1]) or considerably shorter (1:1 lat. inc., 35.4 CI = [32.0/38.8]), this resulted in a lower score. Considerably longer canines resulted in the worst ratings (cf. Figure 2). Regarding the confidence interval and the multiple comparison, “1:1 cent. inc.” and “1:1.05 lat. inc.” showed strong overlap, and thus were rated equally following “1:0.95 cent. inc.”

**Fig. 7:**
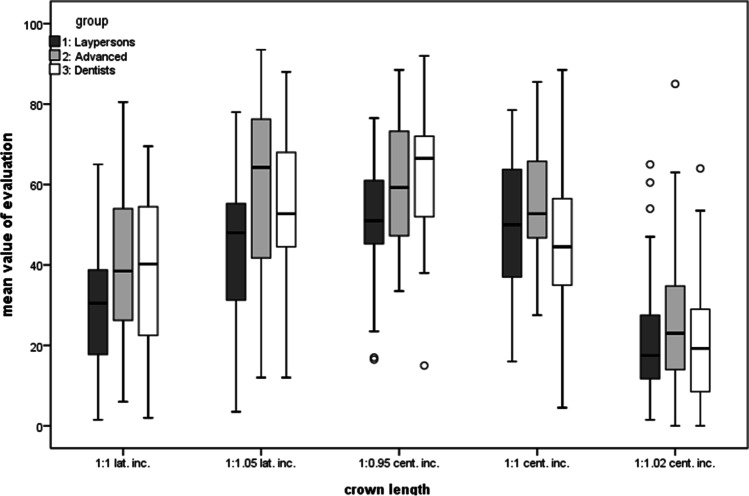
Ratings for the category “crown length of canines”. The ratings show significant differences between length ratios but similar rankings for all groups

The between-group comparison was the only comparison that showed a statistical interaction with the feature category which means that there were relevant differences in the ratings of the three expert groups (*p* = 0.03, see Fig. 7, Table 2).

The cross-group favorite is “1:0.95 cent. inc.,” just as the long canine “1:1.02 cent. inc.” was evaluated worst. Concerning the overlapping confidence intervals, the lay group (group 1) rated “1:1 cent. inc.” almost equally with “1:0.95 cent. inc.” Second place for advanced dentistry students (group 2) and experienced dentists (group 3) was the canine that was slightly longer than the lateral incisors (1:1.05 lat. inc.; group 2, 59.4 CI = [52.7/66.1]; group 3, 54.6 CI = [48.0–61.2]). The group of laypersons/inexperienced dental students (group 1) favored a canine that was as long as the central incisors (1:1 cent. inc., group 1, 50.8 CI = [46.0/55.6]) and evaluated “1:1.05 lat. inc.” correspondingly lower (44.2 CI = [39.1–49.3]).

### Tooth shade

**Fig. 8:**
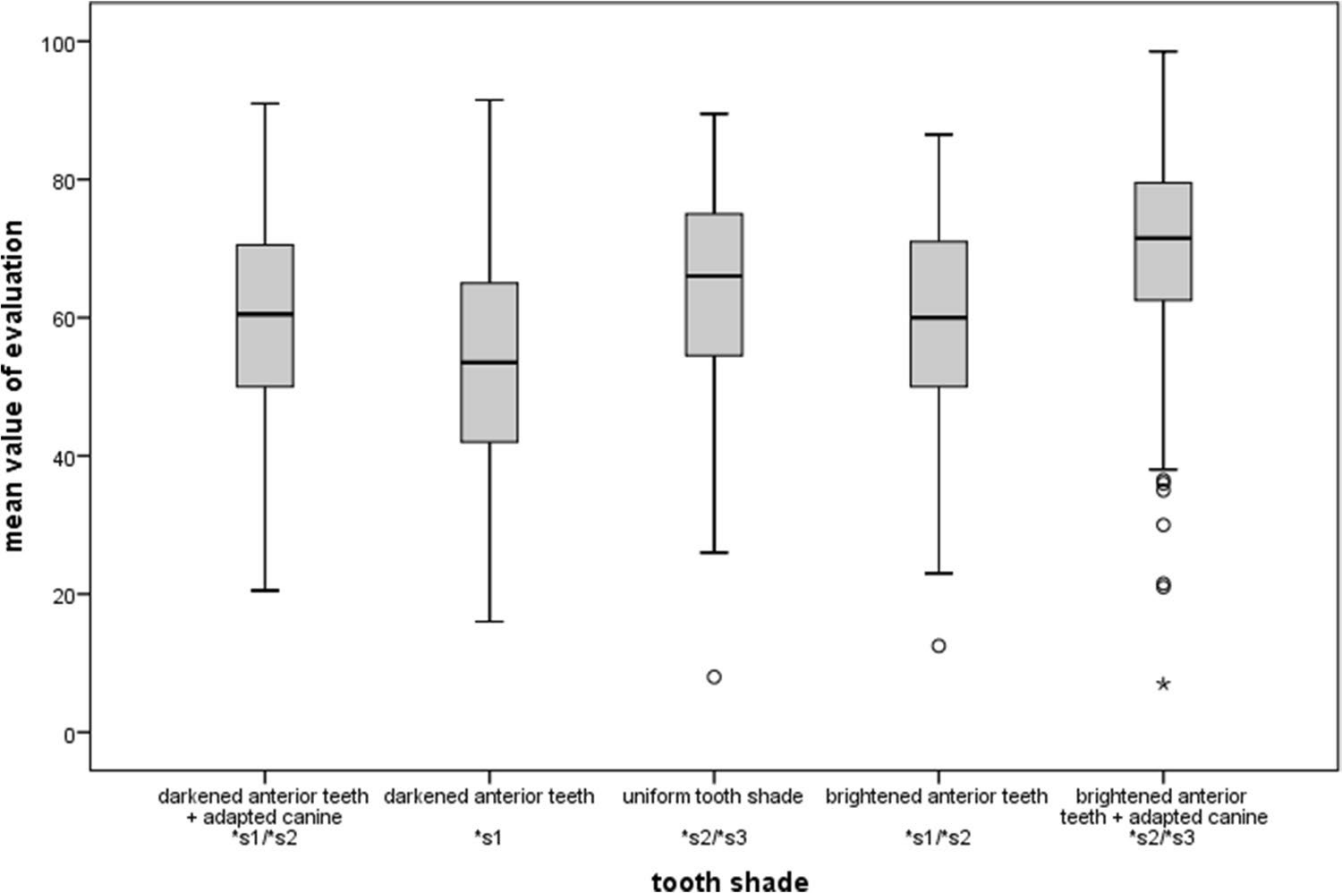
Ratings for the category “tooth shade”. Multiple comparison resulted in three subgroups with *p* ≤ 0.05 across subgroups: The first subgroup is formed by “darkened anterior teeth”, “darkened anterior teeth + adapted canine” and “brightened anterior teeth” (s1). In addition, the last two one form a subgroup with “uniform tooth shade” and “brightened anterior teeth + adapted canine” (s2). These two also form a third subgroup (s3)

As for the tooth shade, an overall brightening of all teeth including the adjusted canine was preferred (67.6 CI = [64.6–70.6]). This was followed by a uniform tooth shade without brightening (63.5 CI = [60.9–66.1]), in which the canine had the same shade as the incisors (see Fig. 8). This type of image modification was rated more esthetic than an overall brighter anterior maxillary region but with natural, i.e., darker, canines (59.3 CI = [56.5–62.1]). Darker incisors generally scored worse. Participants rated a darkened anterior maxillary region with even darker canines worst (53.4 CI = [50.5–56.3], cf. Figure 3). However, if the tooth shade of the canines was adjusted to that of the incisors, i.e., contrary to the natural model (17), this was favored (59.1 CI = [56.3–61.9]). The confidence intervals of this shade modification and brightened incisors without adapted canines showed a large intersection.

Group membership had no significant influence on the evaluation.

### Inclination of canines

**Fig.9:**
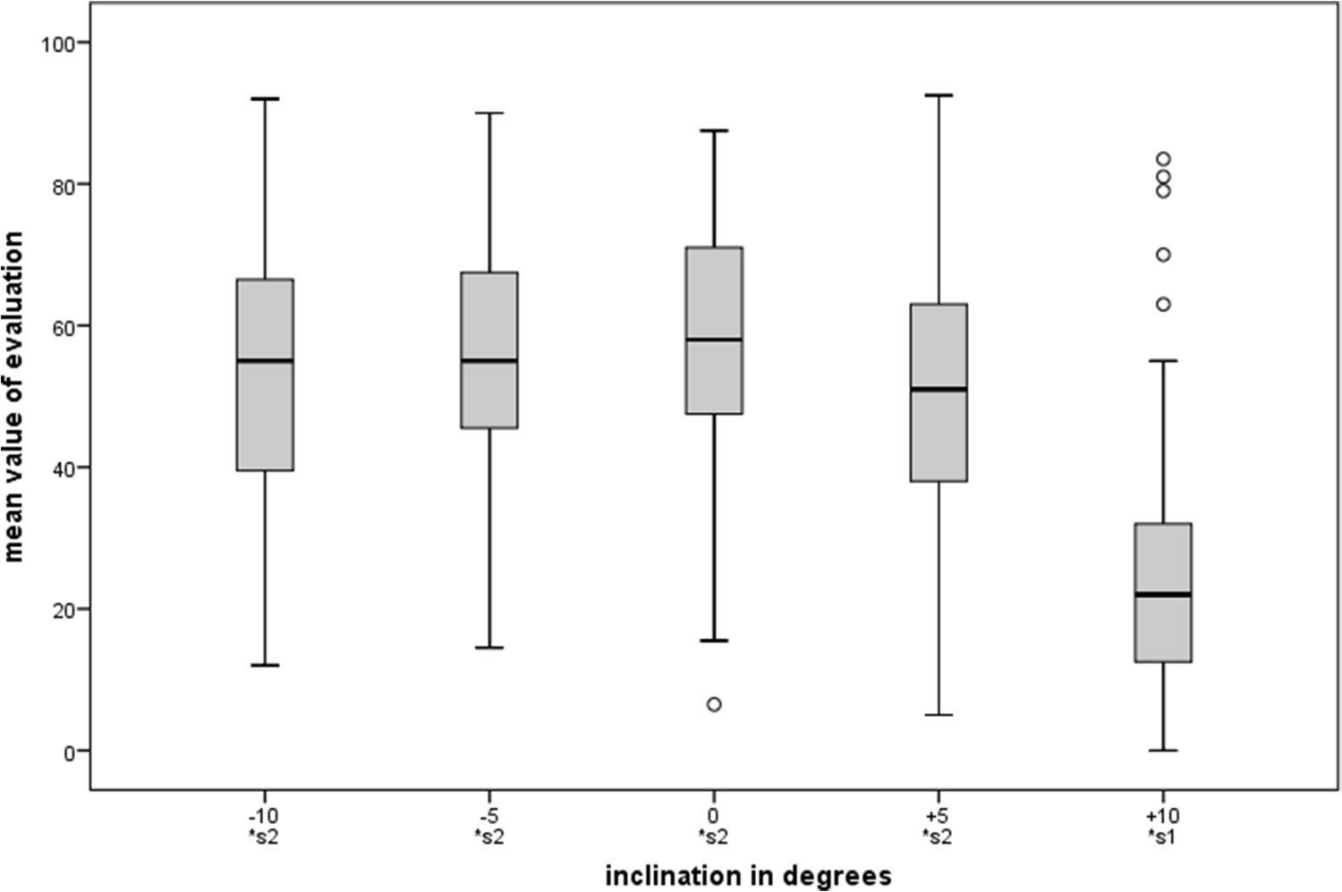
Ratings for the category “inclination of canines”. Multiple comparison resulted in two subgroups: Only + 10° showed significant differences to the other inclinations

A neutral or slightly negative inclination was favored in this category (0° 58.1 CI = [55.4–60.8]; − 5° 56.1 CI = [53.2–59.0], cf. Figure 9). Positive inclinations, i.e., canines that are labially inclined, were consistently perceived and evaluated as considerably less attractive (+ 5° 51.6 CI = [48.6–54.6]; + 10° 23.7 CI = [20.6–26.8]). It should be mentioned that canines with a strong palatal inclination (− 10° 52.7 CI = [49.6–55.8]) were rated only slightly worse on the visual analogue scales than those with an inclination of 0°, which were preferred by most. They were even better rated than canines that have a slight labial inclination (+ 5°, see Fig. 4). The confidence intervals of − 10° and + 5° strongly overlapped. Multiple comparison resulted in a single subgroup for all inclinations from − 10° to + 5°. + 10° which was rated significantly lower.

### Angle of the incisal edge tip

**Fig. 10:**
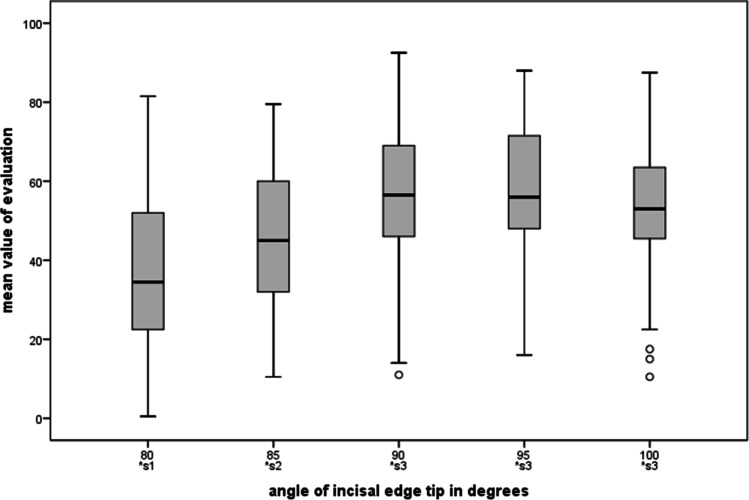
Ratings for the category “angle of the incisal edge tip”. Angles from 90° to 100° form a subgroup; 80° and 85° were rated significantly worse

Overall, acute angles, i.e., incisal edge tips with angles of less than 90°, were rated significantly worse than larger angles (see Fig. 10). On average, an anterior maxillary region with canines whose incisal edge tip was 80° (37.0 CI = [33.6–40.4]) or 85° (46.0 CI = [42.8–49.2]) received low ratings on the visual analogue scales (cf. Figure 5). The opposite “extreme” with an abraded, flattened tip (100° 53.5 CI = [50.6–56.4]) was rated only slightly worse than canines with the preferred angle of 90° (56.5 CI = [53.5–59.5]) to 95° (58.2 CI = [55.4–61.0]). Multiple comparison revealed a common subgroup for 90° to 100°.

## Discussion

Numerous scientific publications focus on oral esthetics. Digitally edited images and photos have often been evaluated [3, 6, 7, 9, 18].

It remains debatable whether the gold standard view for any dentofacial esthetics analysis should be the full-face smile, because these views may represent images of how people interact in a realistic scenario and cofactors such as the soft tissue, the buccal corridor, or the gingival display may influence the overall attractiveness. The face can have a direct impact on the attractiveness of the smile [19]. However, due to natural limitations of viewing a smile (e.g., because of the course of the lip line), an intraoral view must be selected if the main focus is on the canines. Furthermore, intraoral images automatically focus attention on the teeth. Inexperienced observers are thus not unduly distracted by the person’s face or emotions [6, 7]. Influencing cofactors (buccal corridor, gingival display, etc.) were explicitly excluded. For each image, only one quadrant of the maxilla was changed and the changes were then mirrored on the opposite side. In this way, symmetry was ensured, which in turn ensured that the manipulated parameters had the same values on both sides. In this way, asymmetries were avoided, which are perceived as unesthetic and could thus distort the results [5, 20].

In the future, the results of this study should be verified by further studies possibly showing larger parts of the face to exclude that the intraoral view leads to distortion in esthetic perception.

The changes made by the image editing software were relatively small in order to avoid creating striking differences between the individual crown lengths, shapes, and shades (e.g., unnatural vampire teeth). This ensured that study participants had to carefully weigh the attractiveness of the teeth. Since the study was to be as realistic as possible, occlusion and mandibular movement imposed natural limits to digital canine lengthening.

We assembled three groups of participants with different levels of experience in dentistry to evaluate the images. All participants were dentistry students or dentists and were mostly in the same age group. This narrow selection allowed different levels of experience to be accurately defined and verifiably represented. However, it can also be assumed that these participants had both a higher level of education and a pronounced sense of dental esthetics than average. This study could be expanded to include participants from different educational, age, and professional groups to obtain a representative cross-section of society.

Another limitation of the current study is that all participants come from the same cultural background, which may influence esthetic perception. It is conceivable that ideas of beauty are shaped by media and cultural influences. Thus, it does not seem surprising that uniformly bright teeth were evaluated higher in the shade comparison in this study of Central European, Western-influenced participants. This could be one reason why there are no differences between the ratings of the groups. There are ethnic groups, e.g., in Vietnam, where teeth blackening in women is perceived as particularly attractive. However, studies prove a basic cross-cultural agreement on the attractiveness of persons [21, 22].

The use of the visual analogue scales (VAS) allows fine judgment gradations due to the high degree of resolution, which is why image manipulations can also be statistically better distinguished and classified even with high similarity. At the same time, the dimensionless line design without defined categories resulted in increased intra-observer variability in this study. In addition, image manipulation used deliberately avoided extremes, and direct image comparisons were inhibited by the black fades in the slide transition. These factors made evaluation more difficult and thus presumably increased interobserver variability, in addition to a general fatigue effect due to a large number of similar images. Social, mental, or physical competence limitations in completing the VAS as a reason for the variability can be excluded due to the group selection. To reduce the described intraindividual variability, the images were rated twice by the viewers and the evaluation results were averaged.

As described above, the significantly different ratings of the same images suggest that participants suffered from fatigue to some degree. This methodological error could not be eliminated despite providing participants with two short breaks. However, identical rating was unlikely because of the number of images and the use of visual analogue scales without gradation.

Only the photograph of a single smile was used, since the additional retrieval of further smiles would have multiplied the total number of images to be evaluated and would probably have further increased the fatigue effect. For future, more advanced studies with more resources (e.g., higher number of evaluators), the use of multiple smiles seems worthwhile.

Multiple comparisons resulted in different subgroups, allowing the following conclusions: Only acute angles lower than 90*°*, strongly labial inclined, and darker canines compared to rest of the anterior maxillary teeth are esthetically rejected. The crown length has a great influence on esthetics and should vary in a narrow corridor between the lengths of the lateral and central incisors.

Other study groups found similar results regarding canine esthetics and/or the inclination angle [2, 13]. Also preferred inclinations and those rated unesthetic in other studies as well as the lack of significant differences between the evaluations of the different groups of participants were confirmed by the current results. It is noteworthy that only the strongly labial inclination is rated significantly worse and neutral to palatal inclined canines are rated about the same. The fact that comparative studies could show clearer preferences here is probably due to the study design chosen by the other authors (e.g., Q-sort ranking instead of VAS).

With respect to the selected parameters, recommendations for canine morphology can be made based on this study. Since there was an interaction effect in only one feature category and there were thus no significant differences between the groups, it can be concluded that neither experience level nor gender play a role in the ratings. In terms of clinical significance, the morphology recommendations obtained in this study must always be adapted to the individual patient and his or her facial proportions.

## Conclusions

The study allows the following statements.

The canines rated most esthetic by the observers are those that:

1. are almost as long as the central incisor (1:0.95 cent. inc.),
2. have the same shade as the other anterior teeth,
3. are neutral to slightly negatively inclined, i.e., palatal inclined,
4. and have a right angled to rounded incisal edge (≥ 90°).

The least esthetically rated canines are canines that:

1. are longer than the central incisors (1:1.2 cent. inc.),
2. are darker than the other anterior teeth,
3. are positively inclined, i.e., labially inclined,
4. and have tapered incisal edges (≤ 85°).
